# Exosomes as Precise Regulators of the Osteoimmune Microenvironment: Engineering Strategies for Bone Regeneration

**DOI:** 10.34133/bmr.0321

**Published:** 2026-02-06

**Authors:** He Wang, Ruhua Chen, Xinran Li, Jing Wang, Taiying Chen, Shuqi Sun, Xinjie Qiu, Shaobang Wu, Xinyue Zeng, Zhixin Chen, Xiuyun Ren, Bin Zhao

**Affiliations:** ^1^ Shanxi Medical University School and Hospital of Stomatology, Shanxi Province Key Laboratory of Oral Diseases Prevention and New Materials, Taiyuan 030001, China.; ^2^ The Fourth Affiliated Hospital of Guangzhou Medical University, Guangzhou, Guangdong, China.; ^3^ Department of Stomatology, Zhuhai People’s Hospital, Zhuhai Clinical Medical College of Jinan University, Zhuhai, Guangdong, China.

## Abstract

Bone homeostasis is a dynamically orchestrated process that is intricately regulated by the immune system. The emerging field of osteoimmunology has demonstrated that bone homeostasis and repair are governed by a sophisticated crosstalk between immune and skeletal cells, in which immune signals play a critical role in modulating osteogenesis and osteoclastogenesis. Nevertheless, conventional bone repair strategies frequently overlook immune modulation, instead prioritizing structural support or direct osteoinductive effects. Exosomes—endogenous nanovesicles characterized by low immunogenicity and high bioavailability—have emerged as potent mediators within the immune–bone axis. These vesicles mediate intercellular communication by delivering functional cargo, including miRNAs, proteins, and lipids, across biological barriers, thereby enabling precise regulation of inflammatory responses and immune cell polarization. Importantly, exosomes can reprogram the local immune microenvironment from a pro-inflammatory to a regenerative, anti-inflammatory state, thus promoting enhanced bone healing in complex clinical conditions such as osteoporosis and bone defects. This review systematically examines the molecular mechanisms through which exosomes modulate immune responses in bone biology, highlights their pivotal role in reshaping the osteoimmune microenvironment, and discusses their transformative potential in the development of next-generation, precision-based therapeutic approaches for bone regeneration.

## Introduction

Bone tissue is a complex and dynamic system composed of organic and inorganic matrices, cellular components, vascular networks, and immune cells, exhibiting remarkable intrinsic regenerative capacity [1–4]. However, in the presence of clinical challenges such as large bone defects or persistent inflammation that impairs osteogenic differentiation—conditions exceeding its physiological self-repair capability—exogenous bone regeneration materials are frequently required [5–7]. Ideal bone regeneration materials should not only serve as structural scaffolds supporting cell adhesion and proliferation but also possess bioactive functions capable of guiding osteogenic differentiation [8,9].

The development of traditional bone biomaterials has long emphasized direct promotion of osteoblast differentiation [10] while relatively overlooking key pathophysiological barriers at the injury site—such as hypoxia and chronic inflammation—that markedly compromise material integration and tissue reconstruction outcomes [11–13]. Recent advances in bone biology have revealed that successful bone regeneration is not solely dependent on osteogenic precursor cells but rather results from the coordinated interplay of multiple physiological systems, particularly the immune system [14]. This understanding has given rise to the emerging paradigm of “osteoimmunology”, which underscores the pivotal role of the immune system in maintaining skeletal homeostasis and facilitating repair processes [15,16]. In light of this, current research has shifted toward the development of engineered biomaterials with intrinsic immunomodulatory properties [17,18]. The core value of these materials lies in their ability to actively modulate the local immune microenvironment—for example, by promoting macrophage polarization toward the anti-inflammatory phenotype and recruiting regulatory T cells (Tregs)—thereby steering the immune response toward a pro-regenerative state and creating a favorable milieu for bone formation [19,20]. Consequently, the design philosophy of bone biomaterials has evolved from passive “biologically inert scaffolds” to dynamic “immune microenvironment regulators”.

This new paradigm demands innovative tools capable of orchestrating complex multicellular crosstalk with high precision and biological relevance. In this context, exosomes—key mediators of intercellular communication—have garnered substantial attention due to their potent regulatory functions [21,22]. These nanosized vesicles naturally encapsulate and deliver diverse bioactive molecules, including nucleic acids and proteins, traverse biological barriers, and precisely modulate the behavior of target cells, such as osteoblasts, osteoclasts, and immune cells [21,23]. Importantly, exosomes inherit molecular signatures from their parent cells [24], enabling them to orchestrate multicellular crosstalk and exert multifaceted biological effects, including immunomodulation, osteoinduction, and angiogenesis [25]. Their dual or even multimodal regulation of the “immune–bone axis” confers distinct therapeutic advantages in complex clinical settings, such as infectious bone defects and osteoporotic fractures, where concurrent control of inflammation and stimulation of regeneration are essential [26–28]. Furthermore, leveraging the inherent drug-loading capacity and targeting potential of exosomes through bioengineering approaches allows for the precise delivery of specific signaling molecules to bone defect sites, thereby enhancing regenerative efficacy [29].

**Fig. 1.**
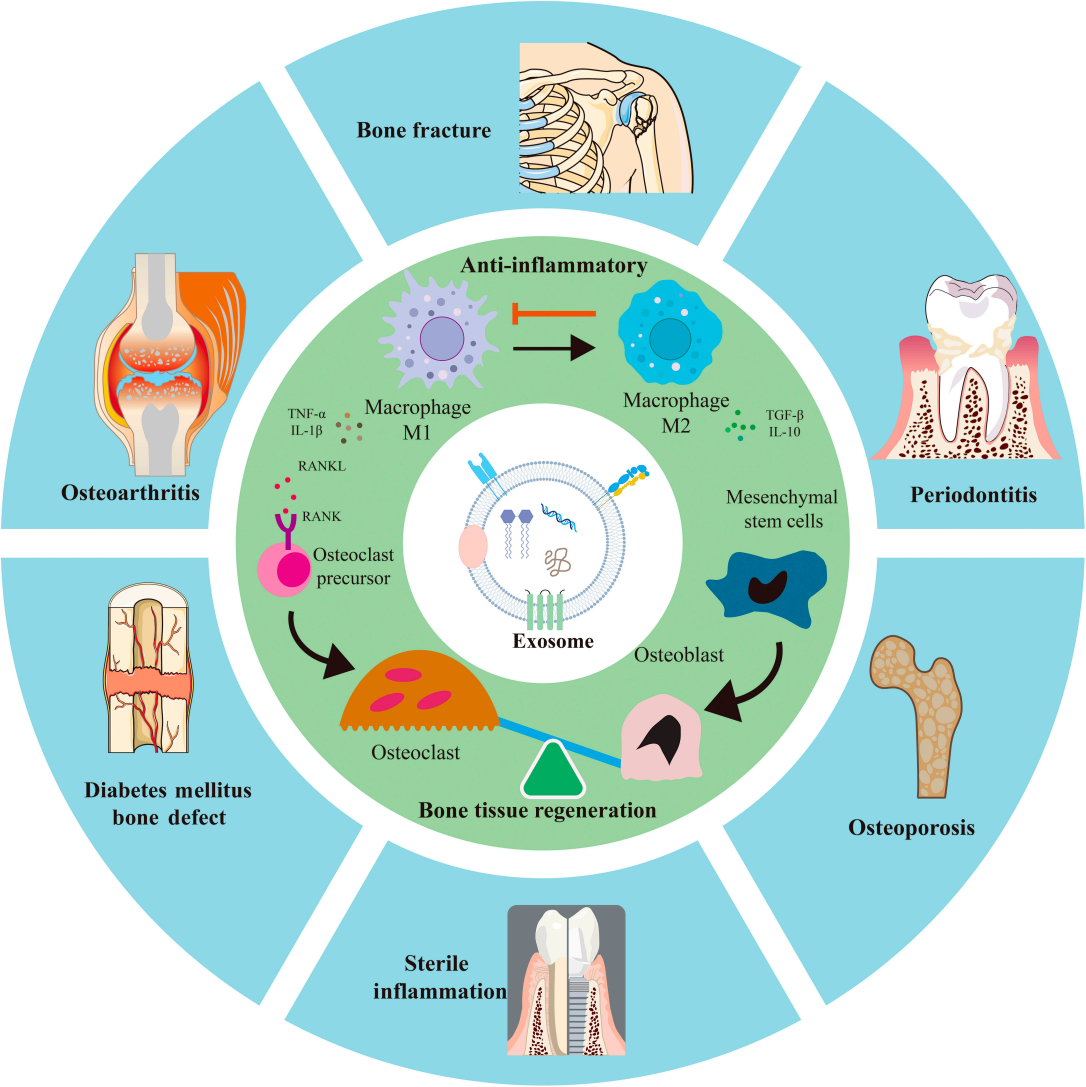
Schematic overview of exosome-based therapeutics through dual “immune-osteogenic” regulation.

Several comprehensive reviews have effectively summarized the broad roles of exosomes in bone regeneration, highlighting their general osteogenic and angiogenic potentials [28,30,31]. Others have provided foundational overviews of osteoimmunology principles or discussed the immunomodulatory potential of biomaterials in a broader context [16,20]. However, a critical gap remains in systematically bridging these domains with a specific and practical focus on exosomes as precise engineering tools for modulating the osteoimmune microenvironment. To address this gap, the present review adopts a unique “engineering-oriented” and “cell-source-specific” perspective. The novelty of the work lies in meticulously analyzing how exosomes derived from specific cellular sources [e.g., mesenchymal stem cells, macrophages in various polarization states, Schwann cells (SCs), and endothelial cells] are intrinsically equipped with distinct molecular cargos that enable them to act as natural, sophisticated delivery systems for reprogramming the osteoimmune microenvironment. Particular emphasis is placed on the dual or multimodal regulatory capacities of exosomes on the “immune–bone axis”, which is paramount for healing in complex pathological conditions like infectious bone defects and osteoporotic fractures, where inflammation and regeneration must be addressed concurrently. This review systematically summarizes the cellular origins of exosomes involved in bone repair, elucidates their underlying molecular mechanisms, and provides a comprehensive overview of advanced exosome-based biomaterial design strategies and their translational potential in clinical applications (Fig. 1).

## Osteoimmune Regulation

**Fig. 2.**
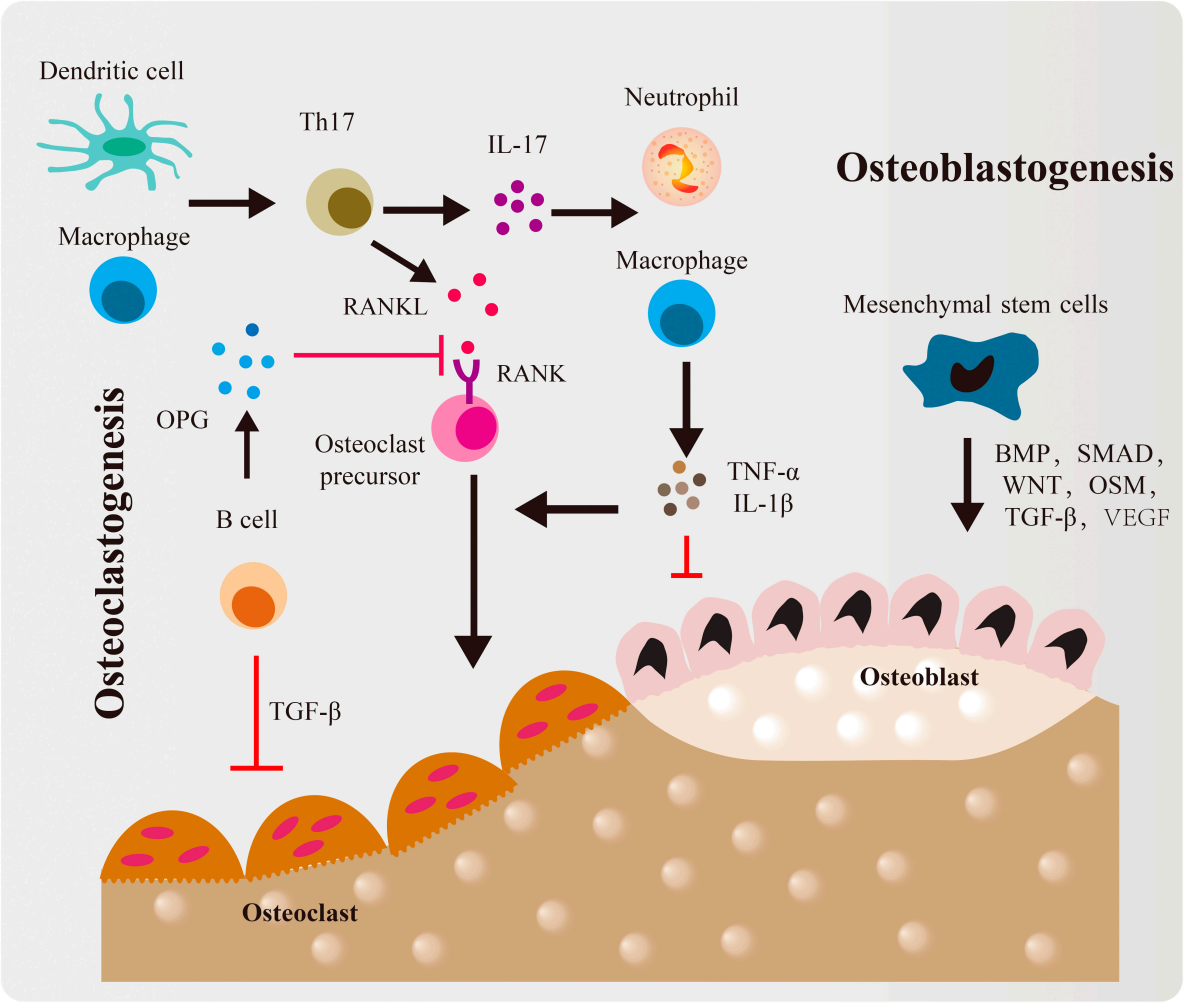
Schematic diagram of immune cell roles in skeletal dynamics. Immune cells actively participate in osteoclastogenesis and osteogenesis by releasing regulatory molecules.

At the beginning of the 21st century, research on osteoclast-activating factors derived from immune cells first revealed the intrinsic connection between the immune and skeletal systems [32,33]. In 2007, Takayanagi [16] formally introduced the concept of “osteoimmunology”, providing a systematic framework for understanding their interactions in bone homeostasis. Studies in this field have established that the immune system regulates bone metabolism through a complex signaling network, wherein cytokines exert decisive effects on the behavior of bone cells (Fig. 2) [34]. Central to this network is the receptor activator of the nuclear factor-κB (NF-κB) ligand (RANKL)/RANK/osteoprotegerin (OPG) pathway [35]. Osteoclast differentiation and activation are dependent on this axis: RANKL, expressed by osteoblasts, osteocytes, and activated T cells, binds to the RANK receptor on osteoclast precursors, triggering downstream transcription factors such as NF-κB and nuclear factor of activated T cells 1 (NFATc1), thereby initiating osteoclastogenesis [36]. Concurrently, OPG, secreted primarily by B cells and other cell types, functions as a decoy receptor by competitively inhibiting the RANKL–RANK interaction, thus preventing excessive bone resorption [37]. The balance between RANKL and OPG is critical for maintaining skeletal homeostasis (Fig. S1A).

The traditional paradigm holds that pro-inflammatory cytokines—including tumor necrosis factor-α (TNF-α), interleukin-6 (IL-6), and IL-1β—primarily promote osteoclastogenesis by enhancing RANKL expression while suppressing osteoblast activity [13]. However, emerging evidence indicates that these cytokines exert more complex and context-dependent regulatory roles in bone metabolism. For example, TNF-α, transforming growth factor-β (TGF-β), interferon-γ (IFN-γ), and IL-17 have been shown to promote extracellular matrix (ECM) deposition under specific conditions, thereby supporting mineralization [38]. This dual functionality arises from the concentration-, time-, space-, and stage-dependent effects of inflammatory cytokines on osteogenic processes [13,34]. Specifically, low concentrations of TNF-α can enhance early bone repair via activation of the NF-κB pathway, whereas high levels inhibit osteoblast differentiation [39]; similarly, low-dose IL-1β promotes mineralization, while elevated levels induce osteoblast apoptosis [40]. Although TGF-β1 contributes to immune tolerance and stimulates osteoblast proliferation, its pro-osteogenic effect diminishes with cellular maturation, giving way to bone morphogenetic protein (BMP)-mediated regulation in later stages of bone formation [41].

Macrophages, key cells of innate immunity, are essential for bone repair. Their function is determined by the balance of 2 polarization states: the M1 phenotype (M1, pro-inflammatory, responsible for initiating inflammation) and the M2 phenotype (M2, anti-inflammatory, responsible for tissue repair and resolution) (Fig. S1B). Although macrophage activation is often described using these 2 categories, accumulating evidence indicates that M1 and M2 merely represent conceptual extremes within a continuous, highly plastic polarization spectrum shaped by microenvironmental cues [42,43]. Recognizing this continuum is essential for accurately interpreting macrophage behavior in bone healing. During the early inflammatory phase, macrophages shift toward the pro-inflammatory end of this spectrum, exhibiting M1-like features such as elevated inducible nitric oxide synthase (iNOS), TNF-α, and IL-6, which initiate debris clearance and regulate early osteoimmune signaling [44,45]. Transient M1-associated factors—including macrophage-derived oncostatin M (OSM)—can even promote early osteogenic events [46,47]. However, prolonged reinforcement of this inflammatory state establishes a chronic catabolic microenvironment that suppresses osteoblast activity, enhances osteoclastogenesis through pathways such as NF-κB, and ultimately impairs regeneration [48]. As inflammation resolves, macrophages gradually transition along the polarization continuum toward reparative, M2-like functional states characterized by IL-10, Arg-1, and other pro-resolution mediators that support angiogenesis, ECM remodeling, and osteogenesis [42,43,49]. Nevertheless, excessive or persistent bias toward the reparative end of the spectrum may promote fibrotic tissue deposition due to elevated TGF-β, platelet-derived growth factor (PDGF), and fibroblast growth factor (FGF) signaling, ultimately compromising functional bone regeneration [49,50]. Importantly, macrophages frequently occupy intermediate or hybrid states rather than residing exclusively at either pole. These transitional phenotypes may coexpress markers associated with both M1- and M2-like states and exhibit a blend of pro-inflammatory and pro-regenerative functions. For example, under specific stimuli—including BMP2 and OSM signaling—macrophages with M1-associated features can also enhance marrow mesenchymal stem cell (MSC) osteogenic differentiation, demonstrating their context-dependent functional plasticity [20,51]. Such hybrid phenotypes play essential roles in orchestrating the temporal progression of inflammatory resolution, vascularization, and subsequent bone formation.

In addition, T and B lymphocytes play essential roles in modulating the bone microenvironment [52]. In autoimmune disorders, dysregulation of lymphocyte subsets profoundly disrupts bone homeostasis [35,53,54]. For instance, IFN-γ produced by T helper 1 (Th1) cells suppresses osteoclast differentiation [55], whereas IL-17 secreted by T helper 17 (Th17) cells up-regulates RANKL expression in osteoblasts and stromal cells while down-regulating OPG, thereby potently stimulating osteoclastogenesis [53,54]. Conversely, Tregs mitigate bone loss by secreting IL-10 and TGF-β, which inhibit osteoclast activity [56]. Beyond their role in producing OPG, B cells also directly support osteogenesis through the expression of Wnt10b and other osteoinductive molecules, underscoring their multifunctional contributions to bone biology [57].

In summary, immune–bone crosstalk is governed by dynamic and spatiotemporally coordinated communication between immune and skeletal cells via intricate signaling networks. A comprehensive understanding of these mechanisms constitutes a fundamental basis for the development of advanced therapeutic strategies for bone regeneration.

## Exosomes

Extracellular vesicles (EVs) comprise a heterogeneous population of lipid bilayer-enclosed particles released by cells, among which exosomes represent a specific subtype derived from the endosomal system and are key mediators of intercellular communication [58,59]. Exosome biogenesis initiates with invagination and endocytosis of the plasma membrane, forming early endosomes (EEs) [60]. Under Rab guanosine triphosphatase (GTPase)-mediated membrane transport regulation, EEs gradually mature into late endosomes, where cargo sorting is completed with the participation of mechanisms such as the endosomal sorting complex required for transport [61]. The limiting membrane of late endosomes undergoes multiple invaginations, forming numerous intraluminal vesicles (ILVs) containing proteins, nucleic acids, and lipids within the lumen. At this stage, the entire endosome is referred to as a multivesicular body (MVB) [60,61]. Some fuse with lysosomes, where their internal vesicles are degraded; others are transported to the plasma membrane under the regulation of proteins such as the Rab family, fuse with the plasma membrane, and release their ILVs into the extracellular space. These released ILVs are exosomes (Fig. S2) [60,62,63]. The molecular composition of exosomal cargo underlies their functional heterogeneity [64]. Exosomes are internalized by target cells via receptor–ligand interactions, endocytosis, and related mechanisms, thereby modulating cellular physiological and pathological processes. Their biological functions span immune regulation, angiogenesis, and bone metabolism, among others [65,66].

In bone-related pathologies, exosome-associated RNA, particularly microRNAs (miRNAs), plays a pivotal role as signaling mediators [67]. For example, in models of inflammatory bone loss, the circulating exosomal miRNA profile is obviously altered, with up-regulation of osteogenesis-inhibitory miRNAs such as miR-125b-5p and miR-214-3p [68,69]. Similarly, in osteoarthritis, subchondral bone-derived exosomes can induce chondrocyte degeneration through the delivery of miR-210-5p [70]. Conversely, exosomal miRNAs with osteogenic or anti-inflammatory potential have garnered increasing interest. Notably, miR-5106 contained in M2 macrophage-derived exosomes (M2-Exos) promotes osteogenic differentiation of bone marrow mesenchymal stem cells (BMSCs) by targeting SIK2 and SIK3 [71]; meanwhile, exosomes carrying miR-1260b have been shown to suppress osteoclastogenesis and enhance M2 polarization [72].

Compared to exosomal RNA, the contribution of protein components to bone regeneration is more complex and remains partially controversial. Exosomes are enriched in tetraspanins (e.g., CD9 and CD63), heat shock proteins, integrins, and other functionally relevant proteins [73]. While some studies report the presence of BMP2 in MSC-derived exosomes and its role in mediating osteogenic effects [74], others demonstrate that BMP2 is undetectable in exosomes secreted by BMP2-overexpressing MSCs, suggesting that the observed osteoinductive activity may instead arise from nonprotein constituents such as miRNAs [75]. These apparently conflicting findings indicate that exosome-mediated bone regeneration likely results from the synergistic action of multiple cargo components. Therefore, the specific functional contributions of exosomal proteins must be rigorously evaluated in a context-dependent manner, taking into account both cellular origin and pathological or physiological conditions.

## Standardized Methodological Practices for Exosome Studies in Osteoimmunology

Recent advances in osteoimmunology have highlighted exosomes as essential mediators of communication between skeletal and immune cells [31]. However, a major limitation across current studies is the insufficient methodological reporting related to exosome isolation, characterization, and dose normalization [76]. Such omissions compromise reproducibility and hinder meaningful comparison across studies. Recognizing these challenges, the MISEV2018 guidelines—and their updated recommendations in MISEV2023—emphasize transparent and standardized reporting of EV-related experimental workflows, including the description of isolation procedures, multidimensional characterization, purity assessment, and explicit dose definition [77,78]. To support methodological rigor in osteoimmunology, we summarize below the key standardized practices, complemented by representative examples from recent exosome studies in bone research.

Exosome isolation remains a critical determinant of purity and functional interpretation. Differential ultracentrifugation (UC) is still the most widely applied method and is deeply embedded in bone-related exosome studies [77]. For instance, TNF-α pre-conditioning adipose-derived stem cell (ADSC) exosomes were isolated through sequential filtration and UC at 100,000*g* for 4 h followed by a second 100,000*g* spin for 60 min, a procedure that aligns well with MISEV-recommended reporting of centrifugal force, duration, and temperature [79]. Similarly, M2-Exos used to induce osteogenic differentiation of BMSCs were purified through UC and subsequently validated for size and marker expression [80]. While UC is accessible and reliable, size exclusion chromatography (SEC) is increasingly favored in studies involving complex biofluids—such as plasma, synovial fluid, or bone marrow aspirates—because it efficiently removes lipoproteins and inflammatory protein complexes that otherwise confound functional assays [81–83]. Polymer-based precipitation, such as polyethylene glycol (PEG)-mediated enrichment, has also been widely used; for example, human adipose-derived MSC-derived exosomes involved in PD-L1-mediated osteoimmune regulation were obtained with a commercial PEG kit [84]. These methods offer convenience but may coprecipitate nonvesicular proteins, underscoring the importance of acknowledging limitations or applying secondary purification (e.g., SEC or ultrafiltration) [85,86]. For studies requiring source-specific exosome enrichment, immune-affinity and microfluidic technologies have proven particularly valuable [87,88]. A notable example is the use of CD39/CD73-functionalized microfluidic chips to isolate highly purified Treg-derived exosomes, which show enhanced capacity to regulate macrophage polarization and promote fracture healing [89].

Consistent with MISEV’s multidimensional characterization framework, exosomes in bone immunology research should be confirmed at the levels of particle size and concentration, morphology, and protein markers [77,78]. Nanoparticle tracking analysis (NTA) is commonly employed to assess particle size distributions within the expected 30- to 150-nm range and to determine concentrations in particles/ml [90,91]. Both TNF-α-ADMC-Exos and M2-Exos were characterized by NTA, while Treg-derived exosomes were evaluated using high-resolution Flow NanoAnalyzer technology, reflecting MISEV2023’s emphasis on improved analytical precision for small exosomes. Morphological assessment by transmission electron microscopy (TEM) remains indispensable; across all reviewed studies—including those using ADSC-Exos, M2-Exos, T cell-derived exosomes, and Treg-Exos—TEM images consistently confirmed intact, lipid bilayer-bound vesicles [71,84,89]. Protein marker analysis using Western blot should include positive exosome markers (CD9, CD63, CD81, TSG101, and Alix) and negative markers excluding contamination from intracellular organelles (calnexin, GM130, or nuclear proteins). Source-associated or functional markers, such as PD-L1 in hADMSC-Exos, Wnt3a in TNF-α–ADSC-Exos, miR-5106 in M2-Exos, or CD39/CD73 in Treg-Exos, can provide additional insight into exosome origin and function but cannot replace core exosome identity markers [71,79,84,89].

Given the strong dose dependence of exosomal effects on osteogenesis, osteoclastogenesis, and immune cell polarization, explicit dose reporting and normalization strategies are essential. Particle number, determined by NTA or tunable resistive pulse sensing (TRPS), is currently the most standardized and comparable metric [92,93]. Total protein content, measured using bicinchoninic acid (BCA) or Bradford assays, is frequently reported—as in studies on ADSC-Exos—but this metric alone may be misleading when isolation methods copurify nonvesicular proteins [79,84,89]. Increasingly, particle-to-protein ratios are used to estimate exosome preparation purity. Dose normalization may alternatively rely on the number of exosome-secreting cells or on the volume of the original biological sample. Crucially, studies comparing exosomes from different cellular origins (e.g., M1 versus M2 and osteoclasts versus osteoblasts) must indicate whether dose equivalence was defined by particle number, protein mass, or producer cell number, as the interpretation of functional potency depends heavily on these choices.

Standardization of exosome methodology is particularly critical in osteoimmunology due to the abundance of cytokines, lipoproteins, and soluble protein complexes in the skeletal microenvironment. Inadequate purification or incomplete reporting may lead to misattribution of biological effects—such as attributing PD-L1 or inflammatory cytokine-mediated responses to exosomes themselves [84]. Likewise, inconsistent exosome dosing can yield contradictory conclusions regarding macrophage polarization, osteoblast or osteoclast regulation, or immune modulation. By adhering to MISEV-aligned methodological practices, future exosome studies in bone research will achieve improved reproducibility, enhanced comparability, more reliable mechanistic insights, and better potential for translational applications in bone regeneration and immunomodulatory therapies. Accordingly, we recommend that all osteoimmunology studies include dedicated sections detailing exosome isolation, characterization, purity assessment, and dose normalization, following the standardized reporting framework outlined in MISEV2018 and MISEV2023 [78].

## Roles of Exosomes from Different Sources in Bone Immunomodulation

The origins of exosomes are highly diverse, encompassing immune cells, bone cells, and stem cells, with their physiological and pathological functions exhibiting marked dependence on cellular source [94,95]. Within the osteoimmune microenvironment, exosomes derived from specific cell types—particularly MSCs and macrophages—have been recognized as pivotal mediators in regulating the dynamic interplay between bone remodeling and immune responses [96]. To elucidate their underlying mechanisms of action, we conducted a comprehensive review of these exosomes and systematically characterized their molecular profiles (Table 1).

**Table 1.** Summary of mechanisms of action of exosomes from various sources in bone homeostasis

Cell source	Mechanism	Cargo	Target cells	Outcome	References
Human mesenchymal stem cells	Mimics the physiological conditions of the bone matrix, the PD-1/PD-L1 axis mediated the increased expression of osteogenic genes, thereby enhancing the osteogenic properties, while the calcium deposits of osteoblasts were maintained.	Exosomal PD-L1	T cells/osteoblasts	Exosomes from hADMSCs can aid in the prevention of bone diseases by maintaining bone homeostasis	[84]
Adipose-derived mesenchymal stem cells	Adipose-derived mesenchymal stem cells reduce the expression of IL-1β and IL-18 in high-glucose-treated osteoclasts by inhibiting the activation of the NLRP3 inflammasome in osteoclasts.	N/A	Osteoclasts	ADMSC-derived exosomes alleviate diabetic osteoporosis by suppressing NLRP3 inflammasome activation in osteoclasts	[115]
Adipose-derived stem cells	Exosomes down-regulate the expression of pro-inflammatory markers IL-6, NF-κB, and TNF-α in activated synovial fibroblasts. Meanwhile, exosomes up-regulate the anti-inflammatory cytokine IL-10, as well as miR-145 and miR-221 in periosteal cells.	N/A	Periosteal cells/synovial fibroblasts	Reducing chronic inflammation and promoting chondrogenesis	[111]
Human gingival mesenchymal stem cells	The results demonstrate that GMSC-Exos exhibits comparable or superior effects to GMSC in suppressing IL-17A and enhancing IL-10 production, thereby reducing arthritis incidence and bone erosion. This mechanism involves inhibiting the IL-17RA–Act1–TRAF6–NF-κB signaling pathway.	N/A	T cells	Reducing incidences and bone erosion	[117]
Human bone mesenchymal stem cells	hBMSC-derived exosome-transferred miR-361-5p alleviates chondrocyte damage and inhibits the NF-κB signaling pathway by targeting DDX20. Inhibition of NF-κB signaling reverses the effect of overexpressed DDX20 on IL-1β-induced chondrocyte damage.	miRNA-361-5p	Chondrocytes	hBMSC-derived exosomal miR-361-5p alleviates OA damage by targeting DDX20 and inactivating the NF-κB signaling pathway.	[118]
Macrophage	Up-regulated osteo-/cementogenic markers and elevated ALP activity; M-Exos activated the p38 MAPK pathway and SB203580 attenuated its promotion effect.	N/A	Human periodontal ligament	M-Exos ameliorated TNF-α-suppressed osteo-/cementogenic differentiation of hPDLCs partly through the p38 MAPK pathway	[126]
M2	Promote the migration, proliferation, and chondrogenic differentiation of bone marrow-derived mesenchymal stem cells	N/A	BMSCs/macrophage	Enhance the repair efficacy of ACECM scaffolds, promote osteochondral regeneration, and regulate the joint cavity inflammatory microenvironment	[168]
Inflammatory macrophage	Up-regulated the expression levels of the inflammatory factors interleukin-6 and tumor necrosis factor-α in BMSCs and mediated inflammatory stimulation; inhibited the transcription levels of the osteogenic genes alkaline phosphatase, type I collagen, and Runt-related transcription factor 2 as well as the alkaline phosphatase activity	N/A	Marrow mesenchymal stem cells	Macrophages in periodontitis can mediate inflammatory stimulation and inhibit the osteogenic differentiation of bone marrow mesenchymal stem cells through the exosome pathway.	[92]
Macrophage	LOC103691165 as a key lncRNA that promoted BMSC osteogenesis	lncRNA LOC103691165	BMSC	M1 and M2 macrophages promoted BMSC osteogenesis by secreting exosomes containing LOC103691165.	[127]
Macrophage	Exosomal miRNA can induce BMSC osteogenic differentiation by directly targeting the SIK2 and SIK3 genes	miRNA-5106	BMSC	The local injection of both a miR5106 agonist or M2D-Exos to fracture sites was sufficient to accelerate healing in vivo.	[71]
Diabetic bone marrow-derived macrophages	Could be transferred into BMSCs to regulate bone regeneration by targeting Smad1	MiR-144-5p	Bone mesenchymal stem cell	Impairs bone fracture healing by targeting Smad1	[80]
B cell	N/A	N/A	Osteoblast and osteoclast	B cell-derived exosomes inhibited bone homeostasis in fracture healing.	[173]
T cell	Runx2, type I collagen, osteopontin, and osteocalcin expression decreased in osteoblasts treated by osteoporotic T cell exosomes	N/A	Osteoblasts	T cell exosomes obtained from osteoporotic and non-osteoporotic individuals could alter the osteoblastic function and gene expression by affecting the genes essential for bone remodeling	[176]
Treg cells	Treg-Exos could effectively catalyze the hydrolysis of ATP and AMP to generate adenosine. With the help of HIF-1α, adenosine would activate adenosine receptors in macrophages and promote M2-like polarization of macrophages	N/A	Macrophages/BMSCs	Treg-Exos and SDF-1 markedly promoted fracture healing	[89]
Human umbilical vein endothelial cells	HUVEC-derived exosomes enable transmitting NEAT1 to alleviate inflammation by inducing M2 polarization of macrophages through DDX3X/NLRP3 regulatory axis	LncRNA NEAT1	Macrophages	HUVEC-derived exosomes promote bone regeneration	[177]

### MSC-derived exosomes

**Fig. 3.**
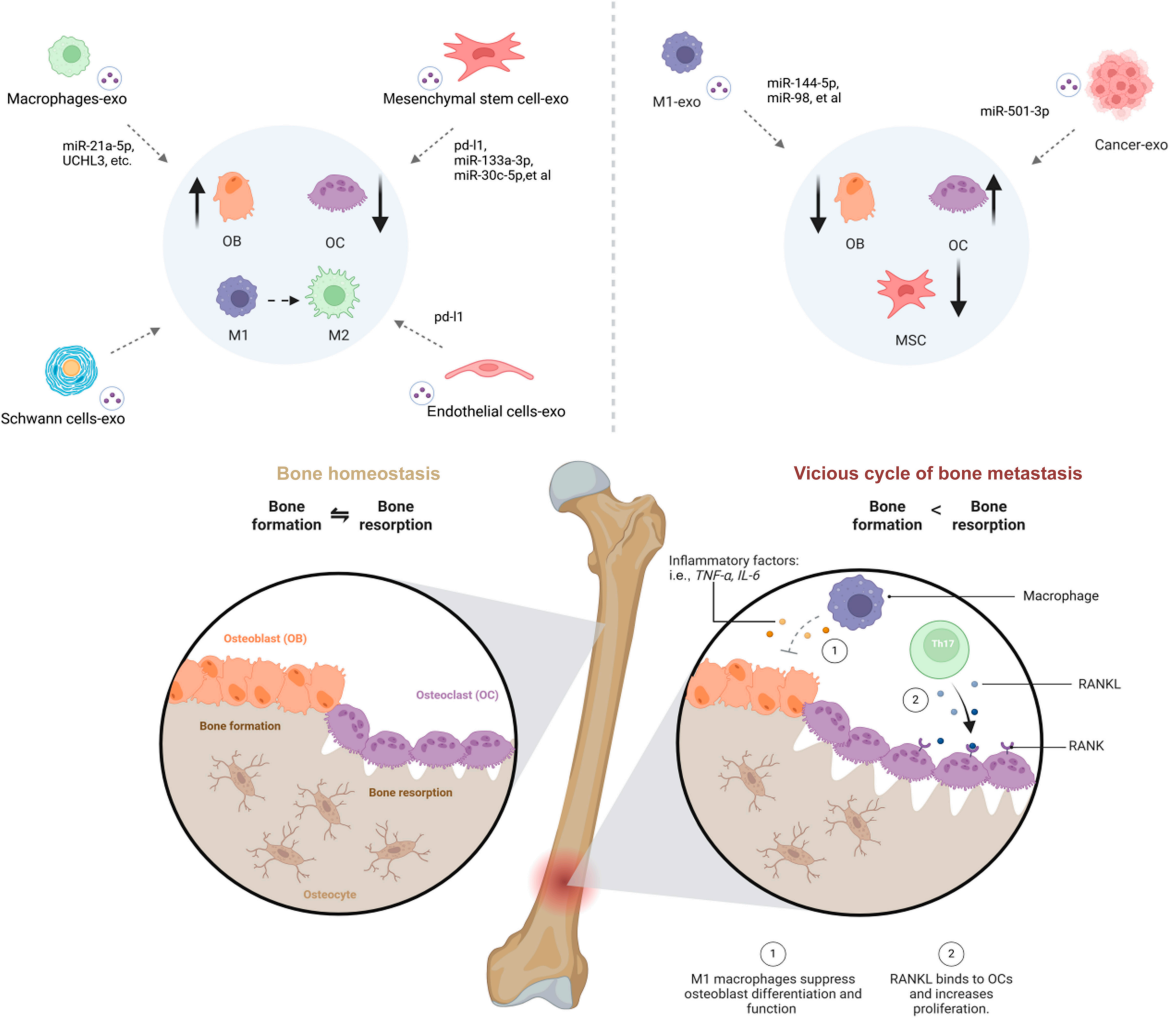
Roles of exosomes derived from different sources in bone homeostasis. Exosomes derived from MSCs, macrophages, Schwann cells, and endothelial cells can increase bone formation and inhibit bone resorption by promoting the activity of osteoblasts, inhibiting the activity of osteoclasts, and promoting the differentiation of MSCs. Exosomes are derived from tumor cells, and M1 inhibit bone formation by inhibiting osteoblast activity, promoting osteoclast activity, and inhibiting the osteogenic differentiation of MSCs. Created with BioRender.com.

MSCs are widely used in cell therapy because of their strong differentiation potential [97]. They can either differentiate directly into functional cells or indirectly promote local tissue growth by secreting bioactive factors [98]. However, challenges such as immune evasion and potential immunogenicity limit their clinical application in bone tissue engineering [99]. MSC-derived exosomes (MSCs-Exos) provide an attractive alternative. These vesicles carry a range of bioactive molecules, transmit key biological signals from their parent MSCs, and induce diverse functional responses in target cells [100]. Applied in bone tissue repair, MSC-Exos exert their effects through 4 major mechanisms: (a) promoting osteoblast differentiation, (b) inhibiting osteoclast activity, (c) stimulating local angiogenesis, and (d) modulating immune responses. The combined action of these mechanisms highlights the strong potential of MSC-Exos as next-generation biomaterials for bone tissue engineering (Fig. 3).

ADSCs, also known as adipose-derived mesenchymal stem cells (ADMSCs), represent a significant category of stem cells extracted from adipose tissue [101]. ADSCs offer advantages such as low immunogenicity, ease of acquisition, and absence of ethical concerns [102]. Given the abundant fat reserves in the human body, a small tissue sample obtained under aseptic conditions yields a large number of stem cells with minimal tissue damage [103]. After separation and purification, these cells can be cultured in vitro with stable proliferation and low mortality rates [104]. Autologous transplantation of stem cells derived from the patient’s own adipose tissue facilitates easy integration with endogenous cells [105,106]. The anti-inflammatory effects of ADSC-Exos have been validated in multiple experiments [107], demonstrating synergistic effects with cartilage/bone protection [108–110] (Fig. S3). When cocultured with synovial fibroblasts, ADSC-Exos modulate the inflammatory milieu by down-regulating pro-inflammatory mediators including IL-6 and TNF-α while enhancing the expression of the anti-inflammatory cytokine IL-10, thereby fostering a tissue-reparative microenvironment [111]. This anti-inflammatory property extends to diabetic osteoporosis models, where these exosomes alleviate bone loss by restraining osteoclast activity through suppression of the NLRP3 inflammasome, leading to reduced secretion of IL-1β and IL-18 [112]. A parallel protective mechanism is observed in models of drug-induced osteonecrosis of the jaw, as ADSC-Exos inhibit the NF-κB signaling pathway, which in turn blocks NLRP3 activation and IL-1β-driven pyroptosis in M1, ultimately supporting alveolar bone regeneration [113]. Beyond immunomodulation, ADSC-Exos directly promote bone and cartilage regeneration. In oxidative stress models, ADSC-Exos enhance expression of chondrogenic markers (e.g., type II collagen and β-catenin) by up-regulating miR-145 and miR-221, while Wnt/β-catenin pathway inhibitors reverse this effect, indicating dependence on classical chondrogenic pathways [111]. Under inflammatory conditions (e.g., TNF-α pretreatment), elevated Wnt-3a content in ADSC-Exos obviously promoted osteoblast proliferation and differentiation, suggesting that the inflammatory microenvironment may further activate the osteogenic potential of ADSC-Exos [114]. ADSC-Exos form a synergistic “immunomodulation–bone regeneration” network by multi-target regulation of the osteoimmune microenvironment (e.g., macrophage polarization and inflammasome suppression) and direct promotion of osteogenic/chondrogenic differentiation. Their functions dynamically adapt to microenvironmental variations (e.g., inflammatory states or disease types).

For immune-related bone diseases like rheumatoid arthritis and osteoarthritis, immunomodulatory exosomes demonstrate precision therapeutic potential [115,116]. For instance, human gingival mesenchymal stem cell exosomes (GMSC-Exos) exhibit precise immunoregulatory capabilities via the IL-17RA–Act1–TRAF6–NF-κB pathway. Compared to parental cells, GMSC-Exos reduced Th17 cell proportions while increasing IL-10 secretion, demonstrating superior efficacy in lowering arthritis incidence and inhibiting bone erosion [117]. Human bone marrow mesenchymal stem cell-derived exosomes (hBMSCs-Exos) deliver miR-361-5p, which alleviates chondrocyte injury by targeting Asp-Glu-Ala-Asp-box polypeptide 20 (DDX20) and suppressing the NF-κB signaling pathway, thereby reversing the detrimental effects of DDX20 overexpression in IL-1β-induced chondrocyte injury and ameliorating osteoarthritis-related damage in vivo [118].

Accumulating evidence supports the regenerative and immunomodulatory potential of MSC-Exos [119]; however, substantial variability has been reported across studies with respect to both the magnitude and, in some cases, the direction of their biological effects, including pro- versus anti-osteogenic outcomes and anti-inflammatory versus neutral or even pro-inflammatory responses [120]. Such discrepancies can be attributed, at least in part, to intrinsic heterogeneity of MSCs themselves—encompassing differences in tissue origin, donor variability, passage number or senescence status, and differentiation stage—as well as to extrinsic factors related to culture and preconditioning protocols [121–123]. Conditions such as hypoxia, inflammatory cytokine priming, acidic or metabolic stress, and serum handling strategies have all been shown to reshape exosome yield and cargo composition, thereby influencing downstream functional readouts [124].

Moreover, emerging evidence suggests that MSC-Exos do not constitute a single homogeneous population but rather comprise functionally distinct subpopulations with divergent molecular signatures and biological activities [125]. Variations in isolation and enrichment workflows may preferentially capture different exosomal subsets, leading to inconsistent functional outcomes across studies and underscoring the need for subpopulation-resolved analyses and standardized methodological frameworks [120].

### Macrophage-derived exosomes

As core regulators of the osteoimmune microenvironment [126,127], macrophage-derived exosomes (M-Exos) deliver functional cargo such as RNAs and proteins to modulate the biological behaviors of immune cells, stem cells, osteoblasts, and osteoclasts [128]. M-Exos play a pivotal role in inflammatory bone diseases, bone defect repair, and implant osseointegration [30,129]. The composition and function of M-Exos vary according to the polarization state of macrophages [130]. Traditional views suggest that M2 dominate immunosuppression and tissue repair, while M1 primarily exert pro-inflammatory effects in the early stages [131,132]. However, their specific osteogenic mechanisms, particularly how they actively promote osteogenesis during early inflammation, remain incompletely understood [20]. Previous studies have demonstrated that macrophages profoundly influence the fate determination of BMSC through paracrine mechanisms, particularly via their secreted exosomes [133]. However, the specific functions of exosomes derived from macrophages in different polarization states and their underlying molecular mechanisms remain controversial and represent gaps in our knowledge.

Pro-inflammatory M1 often play a dominant role in inflammation, secreting pro-inflammatory chemokines to trigger cascading reactions that exacerbate inflammatory responses [45]. The biological signals contained in M1 macrophage-derived exosomes (M1-Exos) are predominantly associated with inflammation induction [134]. Under inflammatory stimuli [e.g., lipopolysaccharide (LPS) and TNF-α], M1-Exos carry proinflammatory factors (IL-6 and TNF-α) and inhibitory miRNAs (e.g., miR-144-5p), which impede BMSC osteogenic differentiation by targeting genes such as Smad1 [80]. Yet, M1 also play a crucial role in early bone healing. M1-Exos not only suppress osteoblast activity but also can promote osteogenic differentiation by activating osteogenesis-related pathways. For example, Liu and colleagues [127] investigated whether M-Exos promote osteogenic differentiation. Results demonstrated that exosomes from both M1- and M2-polarized macrophages enhance BMSC osteogenesis. Specifically, M1-Exos promote osteogenesis in BMSC via miR-21a-5p during early inflammation. Further mechanistic studies revealed that this pro-osteogenic effect is particularly pronounced in M1 and closely correlates with their high expression of the specific miRNA—miR-21a-5p [135,136]. In vitro, M1-Exos accelerate osteogenic differentiation by delivering miR-21a-5p to BMSCs or osteogenic precursor cells (e.g., MC3T3-E1), targeting and suppressing GATA2 expression [136]. Concurrently, exosomes derived from specific macrophage states (e.g., low-inflammatory M1) also effectively activate autophagy pathways within BMSCs. This autophagy activation serves as a key downstream event mediating BMSC migration and osteogenic differentiation [137]. Furthermore, macrophages function as mechanosensitive cells capable of modulating the local inflammatory microenvironment and promoting BMSC osteogenesis through the secretion of multiple mediators. Mechanically induced M-Exos UCHL3 promote alveolar bone formation during orthodontic tooth movement by targeting SMAD1 to enhance BMSC osteogenesis. This elucidates the complex role of M1 in actively participating in osteoimmune regulation during early bone healing [138]. Anti-inflammatory M2-Exos retain the anti-inflammatory properties of M2 [139]. By delivering specific molecules (e.g., miR-365-2-5p and miR-5106) to activate downstream pathways (PTEN/AKT, SMAD1, etc.), they markedly enhance MSC proliferation, migration, and osteogenic differentiation capacity [80]. For instance, miR-365-2-5p promotes MC3T3-E1 osteogenic differentiation by targeting and inhibiting the OLFML1 gene [80]; miR-5106 accelerates BMSC osteogenesis by down-regulating SIK2/SIK3 genes [80]; the long noncoding RNA (lncRNA) LOC103691165 is delivered to BMSCs via exosomes in the fracture microenvironment, activating osteogenesis-related genes (BMP2/RUNX2) [127]. M2-Exos induce M1-to-M2 conversion by stimulating the phosphatidylinositol 3-kinase (PI3K)/AKT pathway. Reducing the proportion of M1 markedly modulates the osteoimmune microenvironment, thereby accelerating fracture healing in diabetes [140]. Although M1-Exos possess immunomodulatory potential, their regulatory mechanisms in bone regeneration remain poorly understood and require further elucidation. In contrast, M2-Exos exhibit more consistent anti-inflammatory and pro-osteogenic effects, making them a more straightforward and promising candidate for use as bone immunomodulatory biomaterials (Fig. S4).

Given the bone immunomodulatory capacity of M-Exos, researchers have proposed strategies to induce M-Exos secretion via biomaterials. DP7-C, a cationic immunomodulatory peptide, induces differential miRNA expression in M-Exos following stimulation, with miR-21b obviously up-regulated. This leads to secretion of miR-21b-rich exosomes. These exosomes enhance osteogenic differentiation in vitro and mitigate periodontal tissue damage in an in vivo experimental periodontitis model. Mechanistically, exosomal miR-21b promotes osteogenesis by directly targeting cytokine-signaling suppressor for interferon γ (SOCS1), thereby activating the Janus kinase 2 (JAK2)/STAT3 signaling pathway [141]. Submicrometer biphasic calcium phosphate ceramics (BCP1) enhance MSC osteogenic differentiation by activating the PTEN/AKT pathway through miR-142a-5p up-regulation in M2-Exos, suggesting that surface morphology design can optimize exosome function.

Inspired by exosome fusion techniques, Ma et al. [142] proposed exosome engineering and combination therapy strategies. Fusing M2-Exos with BMSCs-Exos (M2-BMSCs-Exos) combined M2’s inflammatory targeting with BMSCs’ osteogenic induction capacity, substantially inhibiting periprosthetic bone resorption. The fusion exosome M2-BMSCs-Exos, initially proposed and successfully prepared, provides a new theoretical foundation and solution for the clinical application of therapeutic exosomes.

Despite their favorable immunoregulatory profile, the evidence base supporting the translational potential of M2-Exos in bone repair remains predominantly derived from short- to mid-term preclinical studies, with outcome assessments commonly limited to relatively early time points rather than extended follow-up periods [143]. In vivo studies employing diverse bone regeneration models—including critical-sized cranial defect repair, periodontal bone loss, and fracture healing paradigms—have consistently demonstrated that locally administered M2-Exos exert pronounced osteoimmunomodulatory effects. For example, in critical-sized calvarial defect models, M2-Exos have been shown to promote macrophage polarization toward anti-inflammatory phenotypes, attenuate excessive pro-inflammatory cytokine expression, and establish a regenerative immune milieu conducive to bone formation, often accompanied by enhanced neovascularization within the defect site [144]. In periodontal bone loss and inflammatory alveolar bone defect models, M2-Exos delivery has been reported to suppress inflammatory bone resorption while simultaneously supporting osteogenic differentiation of resident MSCs and improving local bone microarchitecture [145]. Similarly, in fracture healing paradigms—including diabetic or otherwise compromised healing models—M2-Exos administration has been associated with accelerated callus formation, improved vascular ingrowth, and enhanced matrix mineralization, collectively supporting more efficient early-stage bone repair [140]. Across these models, mechanistic analyses commonly implicate M2-Exos-mediated modulation of macrophage polarization, angiogenic signaling, and osteogenic pathways as central contributors to their pro-regenerative effects. However, most studies employ single-dose or otherwise limited dosing paradigms and focus primarily on early regenerative endpoints; systematic dose–response characterization, model-appropriate long-term durability assessments (for example, extended follow-up approaching months in small-animal studies), and comprehensive safety packages remain uncommon [143].

From a biosafety perspective, short-term local administration studies generally suggest low acute immunogenicity and minimal local toxicity under the conditions tested. Nonetheless, clinically relevant translational questions—particularly systemic biodistribution, potential off-target immune modulation, and the consequences of repeated or chronic exposure—are insufficiently defined, consistent with broader challenges in EV therapeutics where pharmacokinetics (PK), tissue tropism, and labeling/quantification artifacts can confound interpretation. Accordingly, describing M2-Exos as a comparatively “straightforward” immunomodulatory modality should be interpreted as reflecting their mechanistic consistency and manufacturability potential rather than established translational readiness [146].

To close these gaps, advancement toward clinical application will require standardized, reproducible preclinical validation frameworks analogous to those used for advanced biomaterials, including (a) batch-to-batch consistency of yield, size distribution, and cargo features; (b) defined storage and stability specifications that preserve potency; (c) quantitative potency assays linking in vitro macrophage polarization and osteogenic readouts to in vivo immune and bone repair endpoints; and (d) PK/pharmacodynamic (PD) studies to define exposure–response relationships and therapeutic durability. Alignment with established EV quality and reporting standards (e.g., MISEV) will be critical to ensure reproducibility, safety, and translational robustness.

Macrophage polarization should not be regarded as a simple binary process but rather as a dynamic and continuous functional spectrum shaped by the nature, intensity, and duration of activating stimuli, as well as by metabolic status and local microenvironmental cues [147]. Accumulating evidence indicates that macrophage phenotypes are distributed along a continuum between the canonical M1 and M2 states, with intermediate activation profiles exhibiting distinct transcriptional, metabolic, and functional characteristics. Within this framework, macrophages residing at different positions along the polarization spectrum are likely to release exosomes with divergent molecular compositions and biological activities, thereby exerting context-dependent regulatory effects on the osteoimmune microenvironment [148].

In principle, investigations of M-Exos in osteoimmunology should therefore extend beyond the 2 extreme polarization states and systematically evaluate exosomes released by intermediate macrophage phenotypes. However, current studies in this field remain largely focused on exosomes derived from the canonical M1 and M2 extremes, using these simplified models to interrogate immunomodulatory and osteogenic functions [143]. While this approach has yielded important mechanistic insights, it may oversimplify the inherent complexity of macrophage biology and consequently limit a comprehensive understanding of how M-Exos orchestrate immune–bone crosstalk across diverse physiological and pathological contexts.

### Other cellular sources of exosomes with potential for osteoimmune regulation

Beyond the aforementioned classical cell-derived exosomes, multiple special-source exosomes participate in bone regeneration through unique osteoimmune regulatory mechanisms, offering novel intervention strategies for complex bone defect repair.

**Fig. 4.**
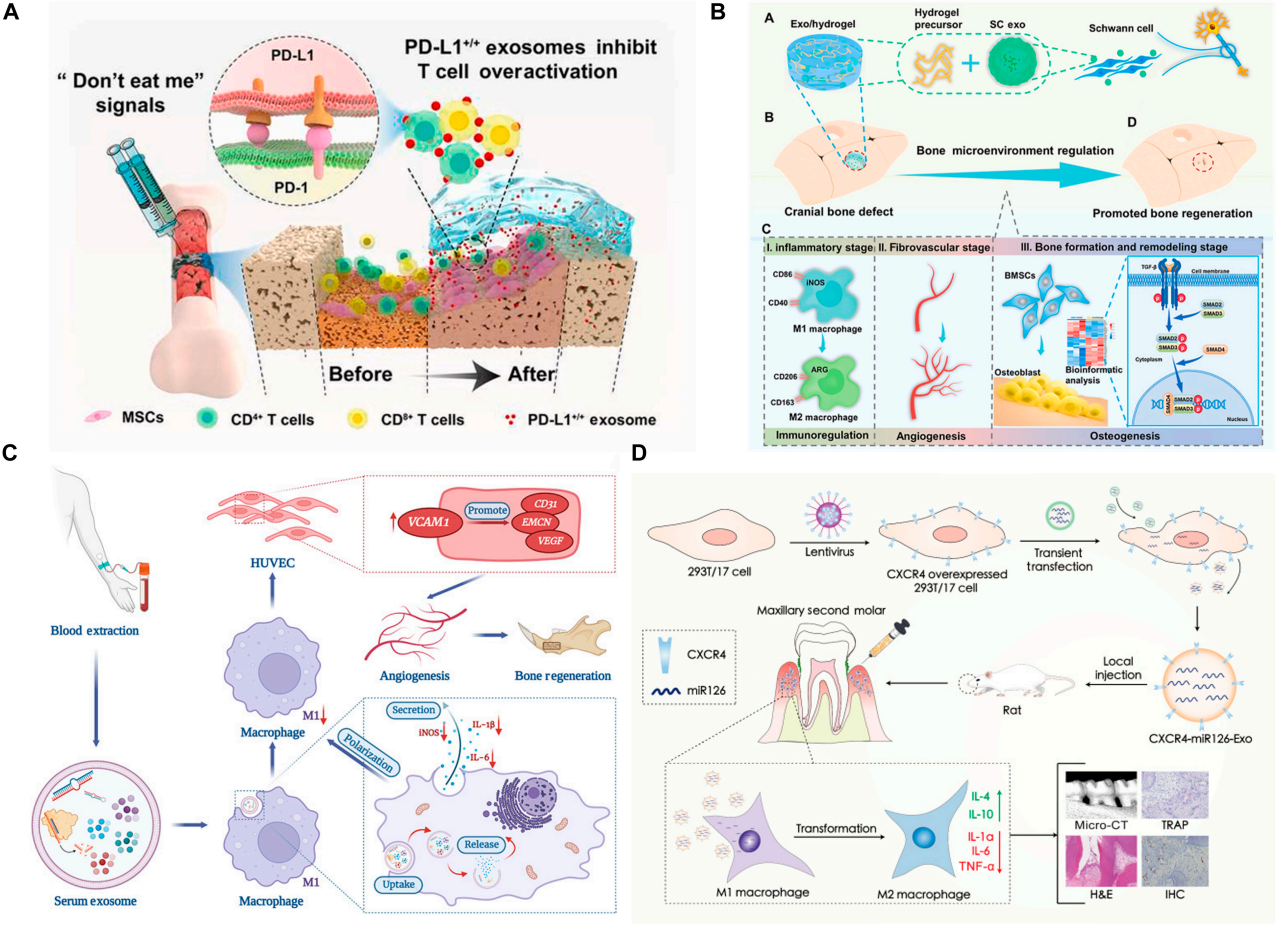
(A) An enriched concentration of PD-L1 was obtained from exosomes HUVECs-Exos. These PD-L1-enriched exosomes function as potent immunosuppressive mediators, capable of attenuating excessive T cell activation through PD-1/PD-L1 signaling. When delivered via an injectable hydrogel system, the exosomes were released in a sustained and localized manner, creating a transient immunosuppressive microenvironment at the fracture site. This immunomodulation promoted osteogenic differentiation and markedly enhanced fracture healing while selectively inhibiting T cell activation in peripheral lymphatic tissues without inducing systemic immunosuppression [149]. Copyright 2021, Elsevier. (B) A multifunctional bone tissue engineering platform was designed based on the bone healing microenvironment, in which SC-Exos were incorporated into an injectable hydrogel system. The sustained release of SC-Exos enables temporal regulation of bone repair through coordinated immunomodulation, angiogenesis, and osteogenesis, thereby promoting cranial bone regeneration [151]. Copyright 2022, Elsevier. (C) Serum-Exos are internalized by macrophages and attenuate pro-inflammatory M1 polarization, leading to reduced expression of inflammatory mediators. The immunomodulated microenvironment enhances VCAM1-mediated endothelial activation and angiogenesis, thereby facilitating vascularized bone regeneration [152]. Copyright 2023, John Wiley and Sons. (D) The CXCR4 modification enhanced exosome homing and uptake by macrophages, promoting macrophage polarization from the pro-inflammatory M1 phenotype toward the anti-inflammatory M2 phenotype, accompanied by decreased expression of inflammatory mediators (e.g., iNOS, IL-1β, IL-6, and TNF-α) and increased anti-inflammatory cytokines (e.g., IL-4 and IL-10). This immunomodulatory effect alleviated periodontal inflammation and bone resorption, ultimately mitigating periodontitis-associated alveolar bone loss [153]. Copyright 2023, Springer Nature.

The proliferation of antigen-specific T cells constitutes a critical immune response to foreign antigens in lymphoid organs. Programmed death-ligand 1 (PD-L1), a transmembrane protein that serves as the ligand for PD-1, inhibits T cell proliferation by transmitting inhibitory signals upon binding to PD-1. Consequently, PD-L1 has emerged as a promising target for regulating excessive immune activation. Recent studies have shown that human umbilical vein endothelial cells (HUVECs) can suppress Treg activation by modulating PD-L1 expression. Exosomes derived from HUVECs (HUVEC-Exos) exhibit the ability to modulate immune activity and participate in bone remodeling. Genetically engineered HUVECs produce PD-L1-enriched exosomes that inhibit T cell activation by binding PD-1 on T cells. Furthermore, when cocultured with T cells, these PD-L1-enriched exosomes promote osteogenic differentiation of MSCs, suggesting that PD-L1-mediated immune modulation contributes to bone regeneration (Fig. 4A) [149].

Human shedding deciduous tooth (SHED)-derived exosomes (SHED-Exos) exhibit significant bone-immune balancing capabilities. In a periodontitis bone defect model, SHED-Exos not only promoted osteogenic differentiation by up-regulating the Runx2/p-Smad5 signaling pathway in BMSCs [an increase in alkaline phosphatase (ALP) activity and mineralized nodules] but also reduced adipogenesis by suppressing peroxisome proliferator-activated receptor γ (PPARγ) expression. Notably, low-dose administration selectively suppressed proinflammatory factors IL-6 and TNF-α [150].

Recent evidence underscores the multifaceted biological requirements for defining the bone microenvironment in bone regeneration. Neurotization holds immense potential for achieving multisystem modulation in bone tissue engineering. SC exosomes markedly enhance bone regeneration by promoting in vivo innervation, inducing M2 polarization of macrophages, HUVEC tubulogenesis, and BMSC osteogenic differentiation. BMSC osteogenesis is primarily promoted through up-regulation of the TGF-β1/SMAD2/3 signaling pathway. Severe periodontitis often leads to irreversible alveolar bone degradation. Periodontal regenerative techniques hold great promise for reconstructing alveolar bone following periodontal disease. SCs play a crucial role in supporting, maintaining, and regenerating periodontal tissues, and SC-derived exosomes (SC-Exos) exhibit cell homing and tissue repair capabilities. However, the specific role of SC-Exos in periodontal bone regeneration remains unclear. To address this, the author treated human periodontal ligament cells (hPDLCs) with SC-Exos and observed significant increases in cell proliferation, osteogenic, and neurogenic differentiation. Furthermore, SC-Exos stimulated the expression of angiogenic factors in vascular endothelial cells. In a rat model of periodontal bone defect, SC-Exos promoted the recruitment of endogenous cells, regulated neuro- and vasogenesis, and accelerated periodontal bone regeneration. This study successfully prepared SC-Exos, which effectively promotes periodontal bone regeneration by modulating the bone healing microenvironment, potentially offering a valuable strategy for periodontal tissue engineering (Fig. 4B) [2,151].

Serum-derived exosomes (Serum-Exos) exert a coordinated immuno–vascular–osteogenic regulatory effect during bone defect repair. Mechanistically, Serum-Exos attenuate M1-associated inflammatory responses and indirectly promote endothelial angiogenic differentiation through macrophage-mediated VCAM1 signaling in HUVECs. This immunomodulation-driven angiogenic activation is characterized by enhanced expression of angiogenic and type-H vessel-associated markers, including CD31 and endomucin, as well as increased microvessel formation. In vivo, local delivery of Serum-Exos obviously enhances angiogenesis and new bone formation in mandibular defect models, as evidenced by increased bone thickness and bone volume parameters, ultimately facilitating coupled vascularization and osteogenesis (Fig. 4C) [152].

Engineered CXCR4-modified exosomes loaded with miR-126 (CXCR4-miR126-Exos) enabled targeted regulation of macrophage polarization in a rat periodontitis model (Fig. 4D). Local administration of CXCR4-miR126-Exos markedly reduced the abundance of pro-inflammatory CD68^+^ macrophages, increased the CD206^+^/CD68^+^ ratio toward an M2-dominant phenotype, suppressed tartrate-resistant acid phosphatase (TRAP)-positive osteoclastogenesis, and obviously attenuated alveolar bone resorption as assessed by micro-computed tomography (CT). These effects were achieved through CXCR4-mediated targeted delivery of miR-126 to macrophages, highlighting the central role of macrophage polarization in bone immune regulation and illustrating a modular exosome-engineering paradigm for the treatment of biofilm-associated osteolytic diseases [153].

## Disease-Specific Contexts in Exosome-Mediated Osteoimmune Regulation

The osteoimmune actions of exosomes are not uniform but are strongly modulated by the underlying disease milieu. Rather than exerting a fixed “pro-regenerative” or “anti-inflammatory” effect, exosome cargo composition, cellular uptake, and downstream signaling are dynamically reprogrammed by systemic metabolic status, chronic inflammation, aging, and defect size [154–156]. Appreciating this disease-specific heterogeneity is essential for rationally designing exosome-based therapies and for interpreting sometimes contradictory findings in the literature.

In osteoporosis and systemic bone loss, exosome signaling often shifts toward a catabolic profile. A seminal study demonstrated that osteoclast-derived exosomal miR-214-3p is transferred to osteoblasts, suppresses osteoblastic gene expression, and reduces bone formation both in vitro and in vivo; osteoclast-specific miR-214-3p knock-in mice exhibit elevated serum exosomal miR-214-3p and impaired bone formation, while osteoclast-targeted miR-214-3p inhibition restores bone formation in aged and ovariectomized models [69]. Data illustrate how exosomes from overactive osteoclasts may consolidate osteoporotic bone loss by reinforcing osteoblast suppression. From a translational standpoint, they also highlight the potential of circulating exosome miRNAs as both biomarkers and therapeutic targets in osteoporosis; however, they raise practical questions about how to selectively modulate osteoclast-derived exosome output without compromising physiological bone remodeling [157–159].

In diabetes-associated bone fragility and fracture nonunion, exosome-mediated osteoimmune crosstalk is profoundly altered. Bone marrow-derived macrophages exposed to a type 2 diabetic milieu secrete exosomes enriched in miR-144-5p, which are taken up by BMSCs and directly target Smad1, thereby impairing osteogenic differentiation and delaying fracture healing (Fig. S5A) [80]. Conversely, exosomes derived from anti-inflammatory M2 have been shown to reprogram the osteoimmune microenvironment in diabetic fractures by decreasing the M1/M2 ratio, dampening excessive inflammation, and accelerating bone repair [136,143]. These contrasting findings underscore a central theme: In metabolic bone disease, M-Exos can function as either deleterious or therapeutic mediators, depending on the polarization state and metabolic imprinting of the parent cell [80,136]. A key translational challenge is therefore to distinguish and selectively harness “pro-regenerative” exosome subsets in a background where endogenous exosomes may be inherently pathogenic.

Inflammatory bone loss disorders such as periodontitis and rheumatoid arthritis provide another paradigm of disease-specific exosome function. The joint and periodontal microenvironments are dominated by chronic activation of NF-κB and inflammasome signaling, skewing macrophages toward M1 and expanding pathogenic Th1/Th17 populations. Gingival mesenchymal stem cell-derived exosomes (GMSC-Exos) have been shown to ameliorate collagen-induced arthritis by suppressing Th1/Th17 differentiation and enhancing Treg responses, thereby reducing joint inflammation and bone erosion [117,160,161]. In this context, exosomes function primarily as immunosuppressive biologics, rebalancing the adaptive immune response rather than directly driving osteogenesis. However, LPS-activated M-Exos in periodontitis and other inflammatory models can deliver cargo that either exacerbates or partially resolves inflammation, depending on their miRNA and protein composition—highlighting significant functional heterogeneity even within a single myeloid compartment [92,162].

Age-related bone loss and skeletal aging introduce further complexity. Recent work indicates that EVs derived from aged bone cells exhibit age-dependent changes in concentration, size, and cargo, with senescence-associated exosomes often carrying pro-inflammatory and pro-resorptive signals that inhibit osteogenic capacity and promote systemic aging phenotypes [163–165]. In contrast, exosomes from young MSCs or osteoprogenitors are enriched in pro-osteogenic and pro-angiogenic miRNAs, supporting bone formation and vascular coupling. This dichotomy suggests that exosomes not only reflect but actively propagate bone aging. Therapeutically, “youthful” MSC-derived exosomes could serve as rejuvenating agents for elderly fracture patients or osteoporosis; however, donor age, comorbidities, and long-term safety of repeated exosome administration remain critical unanswered questions [166,167].

In critical-sized bone defects and osteochondral lesions, successful regeneration requires coordinated modulation of inflammation, angiogenesis, chondrogenesis, and osteogenesis. Here, exosomes are increasingly integrated with biomaterials to achieve spatially and temporally controlled delivery. M2-Exos combined with acellular cartilage matrix scaffolds have been shown to modulate the joint cavity microenvironment, reduce pro-inflammatory cytokines, promote M2, and enhance osteochondral regeneration [168]. Similarly, Wharton’s jelly MSC-derived exosomes loaded within acellular cartilage ECM scaffolds improved osteochondral repair by orchestrating chondrocyte survival, matrix remodeling, and subchondral bone regeneration (Fig. S5B) [169]. These studies highlight that in large defects, exosomes must be considered as part of a composite immunomodulatory–structural system, where scaffold architecture, release kinetics, and local immune status co-determine therapeutic efficacy.

Metabolic and systemic disorders beyond classical osteoporosis and diabetes—such as obesity, chronic kidney disease, and systemic inflammatory syndromes—are increasingly recognized as modifiers of exosome biology in bone. Elevated oxidative stress, uremic toxins, or dyslipidemia may alter exosome biogenesis and cargo loading, reshaping their impact on macrophage polarization, osteoclastogenesis, and endothelial function. Material-based strategies for diabetic bone regeneration, for instance, emphasize not only macrophage regulation and reactive oxygen species (ROS) scavenging but also the potential role of engineered exosomes as adjunctive or synergistic therapies [133,170–172]. However, robust, disease-specific exosome profiling is still limited, and there is a lack of standardized “exosomes signatures” that can guide personalized treatment decisions.

Taken together, these disease-focused observations argue against a “one-size-fits-all” view of exosome-mediated osteoimmune regulation. Instead, exosomes should be conceptualized as context-sensitive immunoregulatory vectors whose effects are shaped by the cellular source, disease state, and therapeutic formulation.

## Conclusion and Future Perspectives

**Fig. 5.**
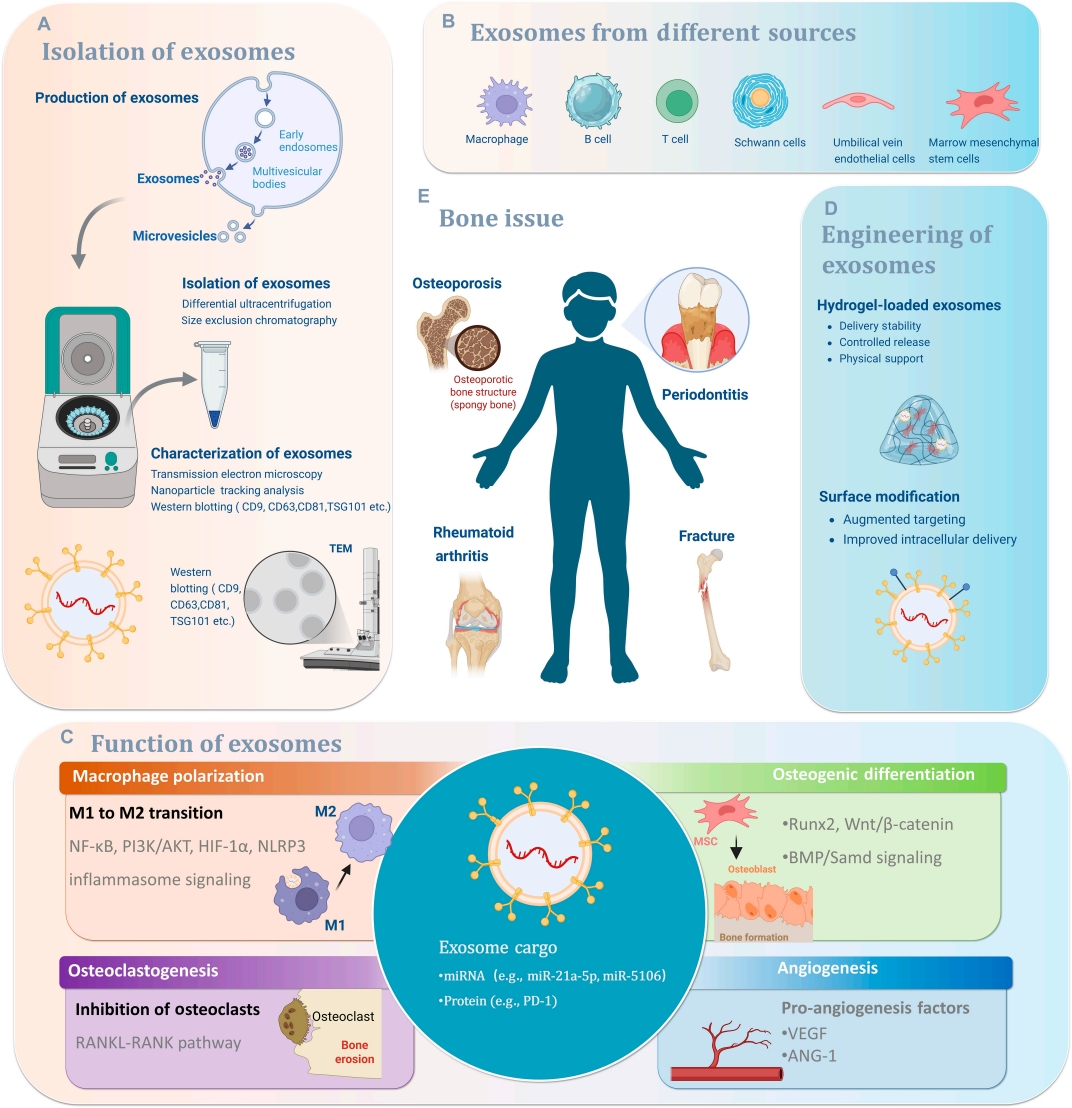
Schematic overview of exosome-based strategies for bone immunomodulation, including exosome isolation and characterization, cellular sources, mechanisms of action, engineering approaches, and therapeutic applications. Created with BioRender.com. (A) Isolation and characterization of exosomes, illustrating their biogenesis, separation methods (e.g., differential ultracentrifugation and size-exclusion chromatography), and standard characterization techniques including transmission electron microscopy, nanoparticle tracking analysis, and marker detection. (B) Major cellular sources of exosomes relevant to bone regeneration, including immune cells (macrophages, B cells, T cells), Schwann cells, endothelial cells, and mesenchymal stem cells. (C) Core biological functions of exosomes in the osteoimmune microenvironment, highlighting their roles in macrophage polarization, regulation of osteoclastogenesis, promotion of osteogenic differentiation, and stimulation of angiogenesis through the delivery of functional cargo such as miRNAs and proteins. (D) Engineering strategies for exosomes, including hydrogel-based delivery systems and surface modifications, designed to enhance targeting efficiency, stability, and controlled release at bone defect sites. (E) Representative bone-related diseases and conditions influenced by exosome-mediated osteoimmune regulation, such as osteoporosis, periodontitis, rheumatoid arthritis, and fracture healing.

Bone regeneration is not solely dictated by osteogenic cells or local biomaterial cues but is instead governed by a dynamic and bidirectional interplay between the immune system and skeletal cells, a concept collectively defined as osteoimmunology. Within this framework, immune cells and bone-resident cells share signaling pathways and regulatory mediators that cooperatively determine the balance between bone formation and bone resorption under both physiological and pathological conditions [16,173–175]. Accumulating evidence further indicates that EVs—particularly exosomes—serve as pivotal intercellular messengers within this immune–bone axis by transferring bioactive molecular cargo that modulates inflammation, angiogenesis, osteogenesis, and osteoclastogenesis. Nevertheless, most existing studies and reviews describe exosome functions in isolated cellular settings or specific disease contexts, resulting in a fragmented understanding and a lack of an integrated mechanistic framework that links exosomal origin, cargo composition, target cells, signaling pathways, and osteoimmune outcomes (Fig. 5) [109,126].

Exosomes involved in osteoimmune regulation are released by a broad spectrum of immune and skeletal cell populations, including MSCs, macrophages with distinct polarization states (M1 and M2), T cells, and endothelial cells [89,119,134,168,176]. Importantly, the cellular origin critically determines the molecular composition, biological activity, and immunomodulatory potential of exosomes. Variations in inflammatory intensity, metabolic status, and tissue microenvironment can profoundly reshape exosomal cargo profiles, thereby conferring either pro-regenerative or pathological signaling properties during different stages of bone repair [80,136].

At the level of key exosomal cargo, these vesicles transport functionally active molecules, predominantly miRNAs, proteins, and long noncoding RNAs, which collectively reprogram recipient cell phenotypes. For example, M2-Exos enriched in miR-5106 have been shown to promote osteogenic differentiation of BMSCs by targeting intracellular kinases involved in osteogenic signaling, highlighting how inflammation-resolving immune cues can be translated into bone-forming signals [71]. In contrast, pathological microenvironments such as diabetes can alter macrophage-derived exosomal profiles, with cargo such as miR-144-5p impairing osteogenic potential and contributing to compromised bone regeneration [80]. In addition to nucleic acids, immunoregulatory proteins—including PD-L1—have been identified within exosomes, underscoring their capacity to simultaneously deliver osteogenic instructions and immune checkpoint signals [149].

Upon release, exosomal cargo is selectively internalized by target immune and skeletal cells, including macrophages, T cell subsets, BMSCs, osteoblasts, and osteoclast precursors, thereby reshaping the osteoimmune microenvironment at a systems level. Despite pronounced heterogeneity in exosomal sources and cargo composition, downstream effects frequently converge on a limited number of core signaling pathways, most notably NF-κB, PI3K/AKT, Smad/BMP, JAK/STAT, and NLRP3 inflammasome signaling. These pathways act as central nodes integrating immune activation, metabolic cues, and osteogenic programs, providing a mechanistic basis for the convergence of diverse exosomal signals [177–181].

Ultimately, through coordinated regulation of immune cell polarization, inflammatory resolution, angiogenesis, osteogenesis, and osteoclastogenesis, exosome-mediated signaling determines the efficiency of bone regeneration and the maintenance of skeletal homeostasis. Notably, these osteoimmune outcomes are highly context-dependent, varying across disease states such as diabetes-associated bone fragility, inflammatory bone loss, and critical-sized bone defects. This context dependence underscores the necessity of an integrative framework that unifies exosomal cellular origin, cargo, target cells, signaling pathways, and functional outcomes to achieve a comprehensive understanding of exosome-mediated osteoimmune regulation and to guide the rational design of future immunomodulatory bone-regenerative strategies.

Exosomes have emerged as a unique class of bioactive nanovesicles capable of orchestrating osteoimmune regulation through dynamic, multidimensional cell–cell communication. Their ability to modulate inflammation, angiogenesis, and osteogenesis simultaneously positions them as a next-generation therapeutic modality for bone regeneration [20,29,96]. However, 3 major barriers continue to limit their clinical translation: (a) intrinsic functional heterogeneity, as exosomes derived from different cell types, polarization states, or disease conditions exhibit substantial variability in cargo and biological activity—for example, diabetic macrophage-derived exosomal miR-144-5p impairs osteogenesis, whereas M2-derived miR-5106 promotes osteoblast differentiation [71]; (b) lack of methodological standardization, with differences in culture conditions and exosome isolation methods (e.g., differential UC, SEC, and polymer precipitation) leading to marked inconsistencies in purity, particle-to-protein ratio, and potency, as highlighted in the MISEV2018/2023 guidelines [77,78,182]; and (c) suboptimal in vivo delivery, as native exosomes are rapidly cleared by the mononuclear phagocyte system and exhibit limited homing to bone tissues [94].

To overcome these barriers, engineering strategies and hybrid material approaches are increasingly regarded as essential for enhancing the therapeutic efficacy and translational readiness of exosomes. Cargo engineering has shown considerable promise: Electroporation-mediated loading of BMP2 mRNA, RUNX2 mRNA, or osteogenic miRNAs can markedly augment the intrinsic osteoinductive capacity of exosomes, while conjugation of pH-responsive peptides enables selective release in acidic inflammatory microenvironments typical of early fracture healing [27,72,74]. More importantly, in complex regenerative scenarios such as critical-sized defects, exosomes must be integrated with 3-dimensional (3D) biomaterial scaffolds to provide structural and biochemical support. Exosome-functionalized ECM scaffolds, bioactive ceramics, and 3D-printed porous constructs have been shown to synergistically modulate macrophage polarization, enhance angiogenesis, and promote coordinated osteogenesis in preclinical models [72,95].

In parallel, scalable production technologies such as 3D bioreactors combined with tangential-flow filtration have considerably improved the batch consistency and yield of exosome preparations, while lyophilization with cryoprotectants (e.g., trehalose) allows long-term preservation without major loss of bioactivity, representing an important step toward the manufacturing needs of good manufacturing practice (GMP)-grade exosome therapeutics [29,183].

A rapidly developing frontier is the engineering of targeted exosome delivery systems, which directly addresses the limitations of in vivo biodistribution. Ligand conjugation—using osteotropic peptides—substantially increases exosome retention in bone-forming regions and enhances uptake by osteoblast lineage cells [184,185]. In addition, chemokine engineering enables exosomes to exploit endogenous migratory pathways: CXCR4-overexpressing exosomes selectively home to SDF-1–rich fracture sites and inflamed tissues, improving immunomodulatory efficacy and accelerating repair [185].

Compared with unmodified natural exosomes, these engineered systems consistently demonstrate greater biological predictability, improved therapeutic index, and enhanced bone-targeting precision, making them strong candidates for next-generation bone-regenerative therapies. Going forward, an integrated design philosophy is likely to dominate the field, combining native exosomal signaling, surface engineering, and smart spatiotemporal release platforms. Such “intelligent exosome–biomaterial hybrids” may ultimately enable precise immunomodulation and physiologic reconstruction of the bone regeneration cascade.

Future research should prioritize 3 directions:

1. Smart activation mechanisms: developing enzyme-, ROS-, or pH-responsive delivery systems tailored to dynamic inflammatory cues at injury sites;
2. Multi-axis integration: designing composite biomaterials that concurrently regulate immune modulation, vascular induction, and osteogenesis, mimicking the coordinated nature of physiological bone healing; and
3. Standardization and regulatory frameworks: establishing comprehensive quality-control pipelines—including batch consistency, cargo profiling, potency assays, stability testing, and PK/PD analysis—aligned with internationally recognized exosome standards to enable clinical-grade translation.

In summary, exosomes represent a paradigm shift from traditional single-factor osteogenic strategies toward microenvironmental reprogramming and biomimetic regeneration. With advances in nanotechnology, genetic engineering, and biofabrication, intelligently engineered exosome platforms hold strong potential to inaugurate a new era of osteoimmune-guided bone regeneration.

## Data Availability

Data sharing is not applicable to this article as no datasets were generated or analyzed during the current study.
