# Supplementary material for: Exosomes as Precise Regulators of the Osteoimmune Microenvironment: Engineering Strategies for Bone Regeneration
Source: Biomater Res. 2026 Feb 6;30:0321. doi: 10.34133/bmr.0321 (PMC12876583; doi:10.34133/bmr.0321)
Supplement: Supplementary 1 — Figs. S1 to S5 Table S1 [file bmr.0321.f1.docx]

**Supplememtary material**


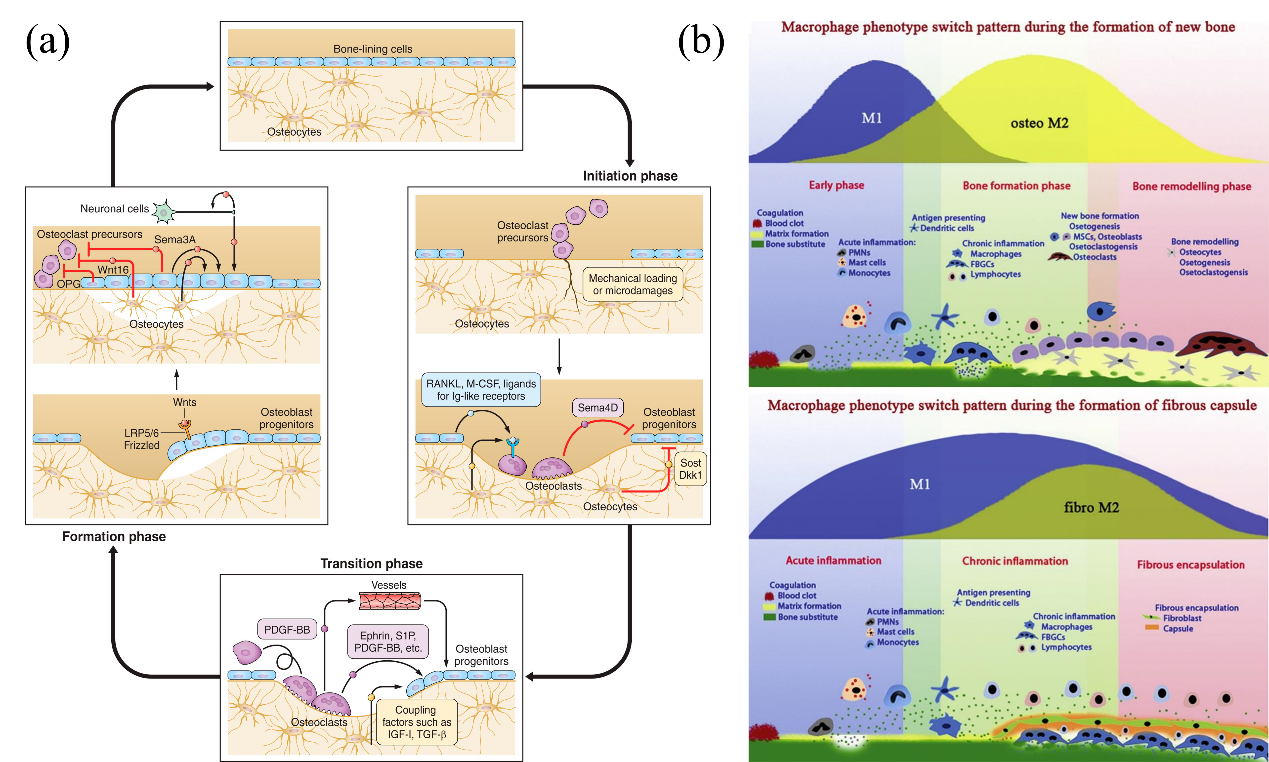


Figure S1 (a) The bone remodeling cycle and bone cell communication factors. The bone remodeling process is divided into the initiation, transition, and formation phases. In the initiation phase, mechanical loading and microdamage are sensed by osteocytes, which stimulate the recruitment of osteoclast precursor cells. Osteoclastogenesis is stimulated by the RANKL, M-CSF, and ligands for immunoglobulin-like receptors, which are produced by osteoblast lineage cells including osteocytes, and bone resorption starts. Osteoclasts inhibit bone formation during bone resorption through the expression of Sema4D. In the transition phase, classical coupling factors, including IGF-I and TGF-β1, stimulate the migration of osteoprogenitors to the resorbed sites and promote differentiation into osteoblasts. In the bone formation phase, osteoblasts replenish the resorbed area with new bone. Sema3A, which is produced by osteoblast lineage cells, inhibits osteoclastogenesis and simultaneously promotes bone formation in this phase[35]. Copyright 2017, American Physiological Society. (b) Schematic of new bone formation mediated by bone biomaterials. The formation of new bone can be divided into three phases: early, bone formation and remodeling phase, with the involvement of multiple systems: [coagulation system](https://www.sciencedirect.com/topics/biochemistry-genetics-and-molecular-biology/coagulation-system), immune system, skeleton system. The most likely macrophage phenotype switch pattern is also presented. The early stage of the repair response is dominated by the inflammatory phase, when the majority of macrophages would be of the inflammatory M1 phenotype. An efficient and timely switch from M1 to [M2](https://www.sciencedirect.com/topics/immunology-and-microbiology/m2-macrophages) phenotype results in an osteogenic cytokine release and with it the formation of new bone tissue. The formation of fibrous capsule is also divided into three phases: acute, [chronic inflammation](https://www.sciencedirect.com/topics/pharmacology-toxicology-and-pharmaceutical-science/chronic-inflammation) and fibrous encapsulation. The likely macrophage phenotype switch pattern is shown. The early stage is dominated by the inflammatory phase, during which the majority of macrophages would be of the inflammatory M1 phenotype. However, a prolonged M1 polarization phase leads to an increase in fibrosis-enhancing cytokine release pattern by the M2 , which results in the formation of a fibrocapsule[20]. Copyright 2016, Elsevier.


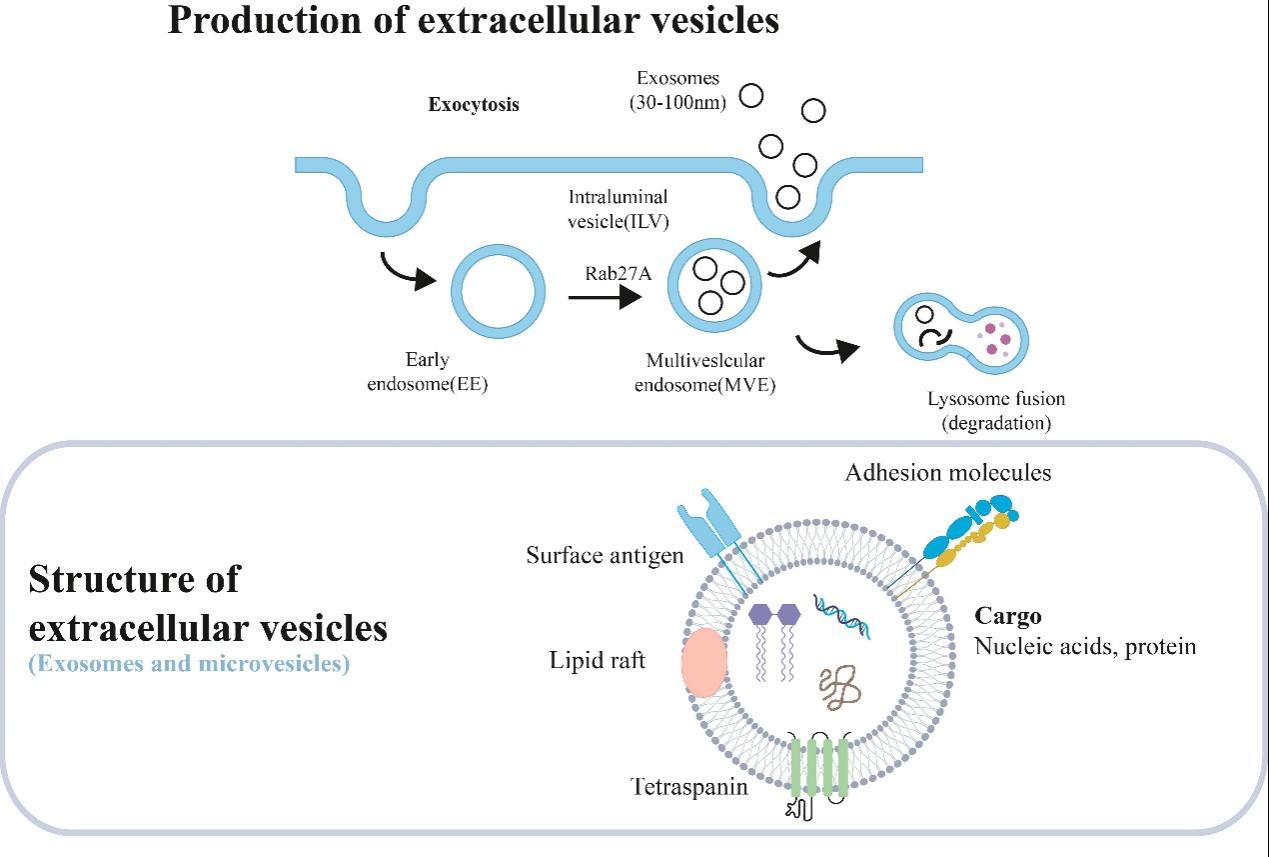


Figure S2 Production and Structure of extracellular vesicle. Following endocytosis, early endosomes (EEs) mature through inward budding of the endosomal membrane to generate intraluminal vesicles (ILVs), leading to the formation of multivesicular endosomes (MVEs). MVEs can either fuse with lysosomes for degradation or, under the regulation of small GTPases such as Rab27A, fuse with the plasma membrane to release ILVs into the extracellular space as exosomes with a typical diameter of approximately 30–100 nm. EVs are enclosed by a lipid bilayer enriched in lipid raft domains and membrane proteins such as tetraspanins and adhesion molecules. Their lumen contains diverse bioactive cargo, including nucleic acids and proteins, enabling EV-mediated intercellular communication and modulation of recipient cell behavior.


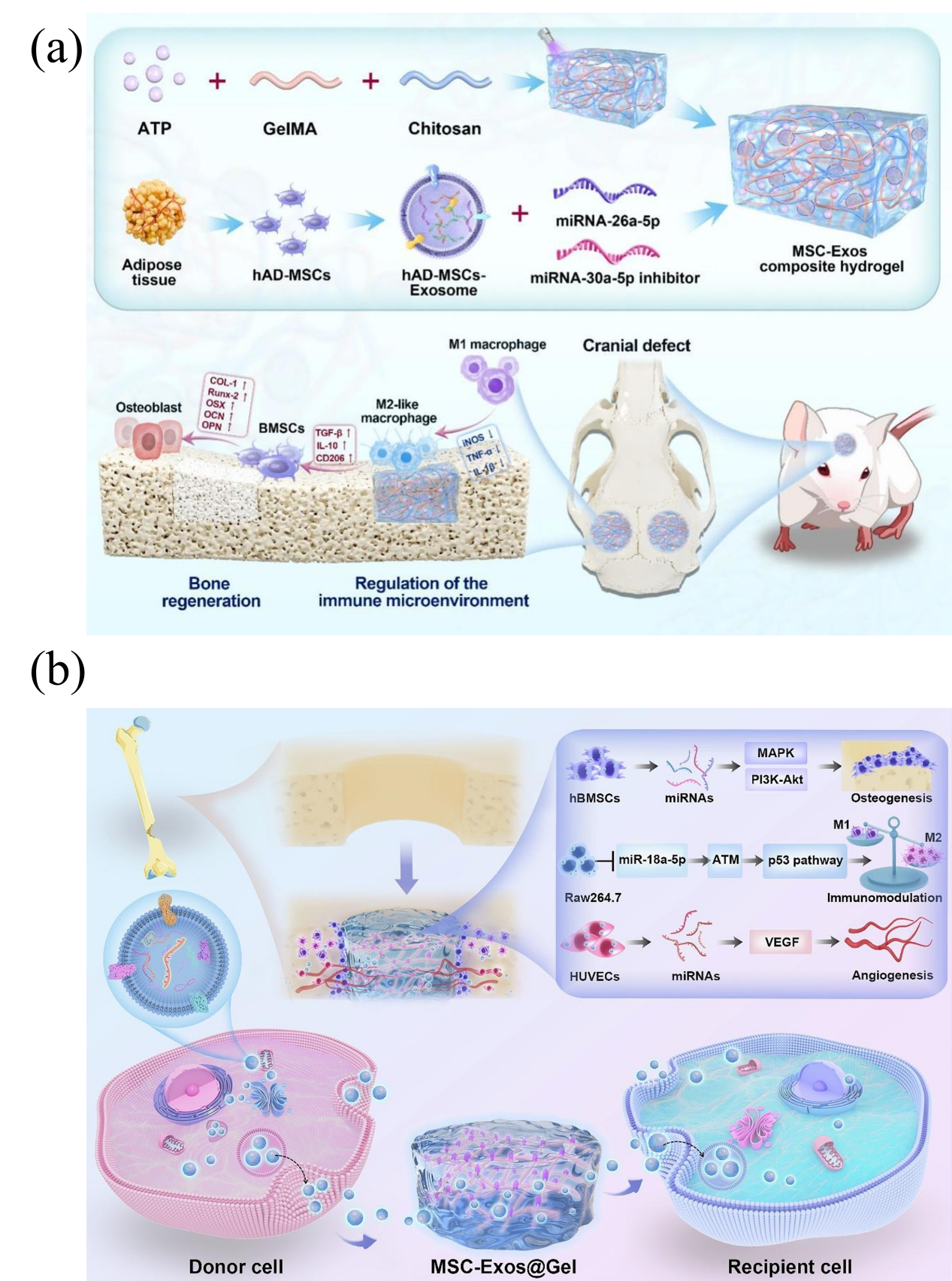


Figure S3 (a) MSC-Exos loaded with miRNA-26a-5p and a miRNA-30a-5p inhibitor were incorporated into an ATP/GelMA/chitosan hydrogel scaffold. The scaffold enables localized and sustained exosome release, promotes M2 polarization, modulates the immune microenvironment, and thereby enhances osteogenic differentiation and bone regeneration in cranial defect models[109]. Copyright 2025, Elsevier. (b) hBMSCs-Exos were encapsulated within a GelMA hydrogel to enable sustained local delivery. The released exosomes transfer bioactive miRNAs to recipient cells, promoting osteogenesis through MAPK and PI3K–Akt signaling in MSCs, inducing macrophage polarization toward an M2 phenotype via the miR-18a-5p/ATM/p53 pathway, and enhancing angiogenesis in endothelial cells through VEGF-related signaling, thereby facilitating coordinated bone regeneration[110]. Copyright 2024, Elsevier.


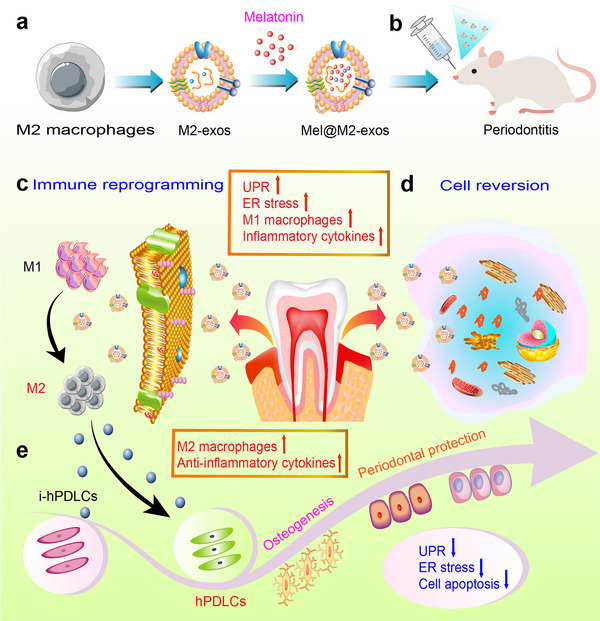


Figure S4 The schematic diagram illustrates the therapeutic mechanism of melatonin-loaded M2-derived exosomes (Mel@M2-exos) in periodontitis. Exosomes secreted by M2 are engineered to carry melatonin and locally delivered to periodontal tissues, where they are taken up by macrophages and periodontal cells. In the inflammatory microenvironment, Mel@M2-exos promote macrophage polarization from the pro-inflammatory M1 toward the anti-inflammatory M2, thereby suppressing endoplasmic reticulum stress and unfolded protein response (UPR) activation and reducing the production of inflammatory cytokines. Meanwhile, Mel@M2-exos directly act on hPDLCs to alleviate TNF-α–induced ER stress and apoptosis, rescuing the impaired osteogenic and cementogenic differentiation. Collectively, these immunomodulatory and cytoprotective effects contribute to enhanced osteogenesis and periodontal tissue protection and regeneration[129]. Copyright 2023, John Wiley and Sons.


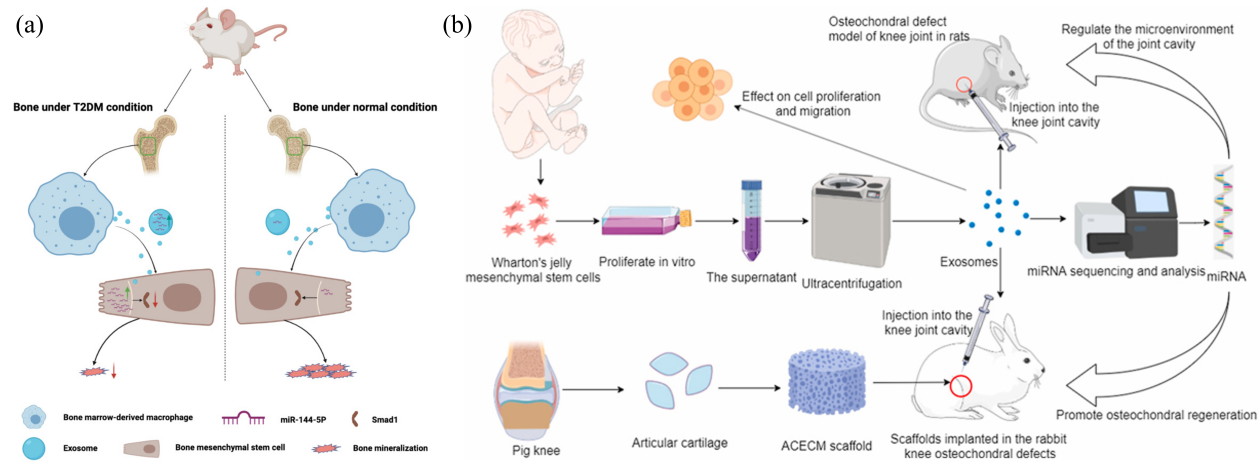


Figure S5 (a) exosomes derived from diabetic bone marrow-derived macrophages-derived exosomal miR-144-5p can be transferred into BMSCs and reduce the bone repair and regeneration by suppressing the expression of Smad1[80]. Copyright 2021, Springer Nature. (b) Human umbilical cord Wharton's jelly mesenchymal stem cell-derived exosomes exert multiple regulatory effects on articular repair microenvironments: they enhance the migration and proliferation of BMSCs, promote chondrocyte proliferation, and induce macrophage polarization toward the M2 to modulate the articular cavity microenvironment[169].Copyright 2021, Elsevier.

**Table S1 Abbreviation**

| **Abbreviation** | **Full Name** |
| --- | --- |
| ACECM | Acellular cartilage extracellular matrix |
| ADMSCs | Adipose-derived mesenchymal stem cells |
| ADSCs | Adipose-derived stem cells |
| Alix | Apoptosis-linked gene 2-interacting protein X |
| ALP | Alkaline Phosphatase |
| Arg-1 | Arginase 1 |
| BCA | Bicinchoninic acid |
| BCP | Biphasic calcium phosphate ceramics |
| BMP | Bone morphogenetic protein |
| BMSC | Bone Marrow-derived Mesenchymal Stem Cell |
| CXCR4 | C-X-C receptor 4 |
| DDX20 | DEAD-box helicase 20 |
| DP7-C | Cholesterol-modified cationic cell-penetrating peptide DP7 |
| ECM | Extracellular matrix |
| EV | Extracellular vesicle |
| FGF | Fibroblast growth factor |
| GM130 | Golgi matrix protein 130 |
| HUVECs | Human umbilical vein endothelial cells |
| IFN-γ | Interferon γ |
| IL-10 | Interleukin 10 |
| IL-17 | Interleukin 17 |
| IL-18 | Interleukin 18 |
| IL-1β | Interleukin 1β |
| IL-6 | Interleukin 6 |
| ILVs | Intraluminal vesicles |
| iNOS | Inducible nitric oxide synthase |
| JAK2 | Janus Kinase 2 |
| lncRNAs | Long Non-Coding RNAs |
| LPS | Lipopolysaccharide |
| MAPK | Mitogen Activated Protein Kinase |
| MISEV | Minimal information for studies of extracellular vesicles |
| MVB | Multivesicular body |
| N/A | Not Applicable |
| NFATc1 | Nuclear factor of activated T-cells 1 |
| NF-κB | Nuclear factor kappaB |
| NLRP3 | NOD-like receptor protein 3 |
| NTA | Nanoparticle tracking analysis |
| OLFML1 | Olfactomedin-like 1 |
| PD-1 | Programmed death-1 |
| PDGF | Platelet-derived growth factor |
| PD-L1 | Programmed Cell Death Ligand 1 |
| PEG | Polyethylene glycol |
| PI3K | Phosphoinositide 3-kinase |
| PK/PD | Pharmacokinetic/Pharmacodynamic |
| p-Smad5 | phosphorylated Smad5 |
| PTEN | Phosphatase and tensin homolog |
| RNA | RibonucleicAcid |
| ROS | Reactive oxygen species |
| Runx2 | Runt-related transcription factor 2 |
| SDF-1 | Chemokine stromal cell-derived factor 1 |
| SEC | Size-exclusion chromatography |
| SHED | Shedding deciduous tooth |
| SIK | Salt Inducible Kinase |
| SMAD | Mothers Against Decapentaplegic Homolog |
| SOCS1 | Cytokine-signaling suppressor for interferon gamma |
| STAT3 | Signal transducer and activator of transcription 3 |
| TEM | Transmission electron microscopy |
| TGF | Transforming Growth Factor |
| Th1 cells | Type 1 T helper cells |
| Th17 cells | Type 17 T helper cells |
| TNF | Tumor Necrosis Factor |
| TRAF6 | TNF receptor-associated factor 6 |
| TRPS | Tunable Resistive Pulse Sensing |
| TSG101 | Tumor Susceptibility Gene 101 |
| UC | Differential ultracentrifugation |
| UCHL3 | Ubiquitin C-terminal Hydrolase L3 |
| VCAM1 | Vascular Cell Adhesion Molecule 1 |
